# Documentation framework for healthcare simulation quality improvement activities

**DOI:** 10.1186/s41077-017-0053-2

**Published:** 2017-10-17

**Authors:** Melanie Barlow, Robyn Dickie, Catherine Morse, Donna Bonney, Robert Simon

**Affiliations:** 1 Mater Education Ltd, Queenslane, Brisbane, Australia; 2 Center for Medical Simulation, Queenslane, Brisbane, Australia

**Keywords:** Quality improvement, Latent errors, Latent threats, In situ simulation, Systems testing, Facility testing, Plan-do-study-act, Healthcare failure mode effects analysis, Point of care

## Introduction

Medical simulation methodology is increasingly being utilised beyond the traditions of education to evaluate patient care workflows, processes, and systems within the health context. A literature review of healthcare facility testing showed that individual clinical departments and singular patient flow processes had been tested under a variety of simulated conditions, such as virtual environments, table top exercises, and live simulation exercises. Each method demonstrated strengths and weaknesses in finding active or latent system failures [1–4]. With the building of our new healthcare facility, it was decided that live (physical) testing of the environment using a medical simulation methodology was the best approach to bridge the gap from architectural plans, to real-world efficient and effective patient care, and for orientation and training of teams to their new environment [4–7]. Although the hospital had yet to open, testing the systems under immersive simulated conditions at the point of care delivery effectively replicated real-world workflows and systems [1, 6]. Within Australia, two new hospitals reported using medical simulation to test specific clinical scenarios and patient flow journeys prior to service delivery. Unfortunately in both instances, testing beyond the first round did not occur due to funding and human resource limitations. This led to considerable staff workarounds, rectification of process errors *after* commencement of patient care, and unfavourable media reports [8, 9].

This paper will provide an example of an approach to identify latent system issues using live medical simulation and the development of an associated documentation framework. The documentation framework aims to help structure medical simulation scenarios specifically designed for quality improvement activities, and to capture and report findings of system deficits identified in the simulations, to key decision-makers.

Our metropolitan mixed public and private healthcare organisation built a satellite health service outside the capital city specialising in day oncology and day surgery, with 64 short stay surgical and medical inpatient beds. Two significant dilemmas were apparent: over half of the staff were new to the main organisation and no onsite critical care support was available. Additionally, the new facility was adapting existing processes from the main central facility, where services were not similar. A serious potential risk to patient safety was noted. Organisational priorities for opening included efficient and effective staff training and systems designed to ensure patient safety in concert with excellent patient experience. Testing of a whole healthcare facility is a large undertaking. It was decided that testing needed to occur over multiple iterations, allowing for system improvements to be made and retested. The final testing cycle was a 24-h live simulation activity. A critical part of the activity was data collection, which led to the development and utilisation of two new tools: the Simulation-based Quality Improvement Tool (SQIOT) and the Healthcare Failure Modes Effects Analysis (HFMEA) Summary Report.

The first reporting tool, SQIOT, utilised the Plan-Do-Study-Act (PDSA) methodology [10–12] as the scenario template to capture data arising from each simulation activity. A second tool, ‘HFMEA Report Summary’, was underpinned by the Healthcare Failure Modes Effect Analysis (HFMEA) framework [13, 14]. The HFMEA framework provided a way to collate the data and to target summary data to accountable leaders. The combination of the PDSA and HFMEA frameworks as developed and described in this article is a previously unidentified strategy in the literature. The overall design of the simulation activity and individual scenarios are outside the scope of this paper. Table 1 outlines the life cycle of the project. Following Table 1 is a detailed description of each phase of the respective tool development.

**Table 1:** Four phases of implementation over the life cycle of the project

Phases/location	Date 2015	Actions
Phase 1 Planning and preparation *Simulation centre*	May–Sept.	a) SQIOT drafted as scenario template and for data collection during iterative simulations b) HFMEA Report Summary drafted to present collated and risk-rated data from the simulations to decision-makers. c) Scenario development and instrument testing undertaken within the simulation centre: face validity. Key action Phase 1: testing of *documentation tools* and *scenarios* within the simulation centre
Phase 2 Simulation testing *In situ*	Oct. 1, 2 Oct. 6, 7	Test 1: ‘In-hospital’ simulation testing of systems and workflows with hospital leaders and senior clinicians. Identification of required amendments to systems and workflows. Scenarios refined based on revised processes. Test 2: ‘In-hospital’ simulation testing of systems and workflows with hospital leaders and senior clinicians repeated. Identification of further required amendments to systems and workflows. Scenarios refined based on revised processes for Phase 3. Key action Phase 2: Testing of *documentation tools*, *scenarios and processes/systems/workflows* within the hospital.
Phase 3 Simulation testing *In situ*	Oct. 22	‘24-h’ simulation in situ simulation testing with hospital staff and simulated patients. Key action Phase 3: testing of *processes/systems/workflows* within the hospital over a consecutive 24-h period.
Phase 4 *In situ*	Oct. 23 Oct. 28	‘Post-simulation’ staff survey conducted by hospital leadership: 100% (*n* = 112) participating staff confident to open hospital. Report Summary (HFMEA) submitted: Phases 2 and 3 data highlighting improvements made and further recommendations for refinements to processes and workflows. Hospital opened with commencement of full service delivery.

### Simulation activity Phase 1: planning and preparation

Objectives:

1. Identify existing documentation tools designed to test healthcare systems and workflows using live simulation methodology
2. Identify existing documentation tools designed to report outcomes of the systems and workflow simulations to key stakeholders
3. Design appropriate in situ simulation scenarios to test systems and workflows

The predominant determinant for operational readiness was patient and staff safety. This was due to the many interdependencies between the physical environment, the processes, and the people (human factors) [15]. It was identified early that accurate and easy-to-use documentation tools to capture the identified concerns was an essential element of the project. A review of the literature did not reveal previously used documentation tools in healthcare to support facility and system quality improvement activities. It was decided that the team needed to devise its own tools for simulation delivery, data collection and data collation. As many of the patient flow processes were still in draft form at the time of initial testing, it was anticipated that there would be a significant number of observed gaps in the processes and a number of clinical concerns raised by end users during the systems and workflow tests. The Mater Education Simulation Team (MEST) led the execution of this large-scale medical simulation test. Three questions that the MEST aimed to address were:

1. How to document the simulations as a system and workflow test in comparison to traditional educational simulations?
2. What would be the best method to collect and collate the large data sets in the context of concurrent, iterative simulations?
3. In what format should the data be presented to the organisation’s decision-makers to ensure that staff would be prepared and oriented, and that the systems and processes are safe?

The Plan-Do-Study-Act (PDSA) methodology provides a structure for cyclical or iterative testing of changes to a system or process, to improve quality and effectiveness [11, 16–18], and therefore ideally suited the iterative nature of our activity. There was little in the literature about using the PDSA framework for quality improvement within medical simulation [6, 7, 19, 20], and there was no evidence of a standard template for running medical simulation scenarios specifically to test clinical and non-clinical processes. As a result, the MEST developed the SQIOT.

### Simulation-based Quality Improvement Observation Tool (SQIOT) Design

Usually, PDSA Quality Improvement (QI) activities evaluate existing clinical processes when a process change has been implemented and needs to be assessed. The PDSA process aims to identify a distinct relationship between an intentional change in a process, and any variation (positive or negative), to the intended outcome [16]. A complexity with this activity was that the majority of existing procedures, patient flow, staff workflow, and emergency response processes were new and untested. This meant that the more focused use of the PDSA methodology could not be utilised. Instead, each process or system was initially assessed as a whole, with all strengths and weaknesses documented, and system amendments then made for the subsequent testing cycles. The benefit of the PDSA methodology was that it provided the required flexibility for testing within this context [10, 11].

**Fig. 1:**
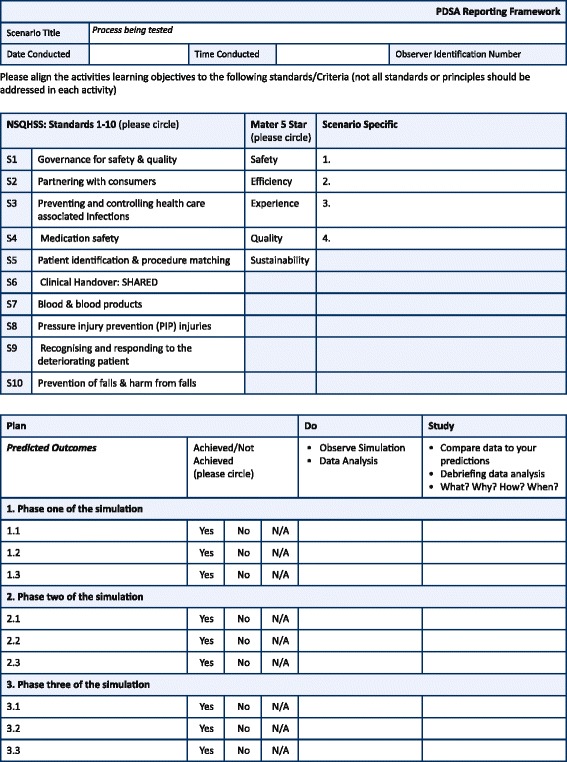
Sample section from the final version of SQIOT (pages 1–2 of the form)

The required data to be captured included identified hazards, latent failures, concerns surrounding the patient experience, and suggested quality improvements; most of which could not be pre-identified with specificity. The SQIOT design offered a template that focused on system integrity and quality improvement. It allowed data to be captured from different sources, at different intervals and via different methods, such as direct observations and post-simulation debriefings. The debriefs occurred after every simulation scenario, in each phase (Phases 1–3), and included participating staff, observers, external providers, and simulated patients. Trained simulation faculty facilitated the debriefings. Additionally, a large debrief occurred in Phase 4 with all staff, external providers, and simulated patients who were present at the conclusion of the 24-h simulation event. The SQIOT was organised using the PDSA methodology. Each section is displayed in Fig. 1 and described.

#### Plan

The ‘Plan’ component in the template was divided into a number of elements. The front pages were derived from the organisations’ simulation scenario template. It outlined simulation objectives; key scenario information orientating people to time, place, situation, and alignment to organisational strategy; and national safety standards [21]. The larger corporate organisation’s five strategic priorities for its business were Safety, Efficiency, Future, Quality, and Experience [22]. Alignment with the organisational strategy was essential for leadership engagement, support in the form of human and financial resourcing, and their commitment to support the required actions when failures were identified. Additionally, alignment to the Australian National Safety and Health Quality Service Standards (NSHQSS) [21] assisted the organisation in the attainment of robust evidence for hospital accreditation. The ‘Plan Phase’ within the tool documented each step of a drafted individual process or workflow, which acted as the basis for scenario script.

#### Do

The ‘Do’ component was the execution of the simulation activity, wherein the observers of the simulation identified if a specific step in the process was or was not achieved by circling ‘yes’ or ‘no’. Data was also captured through free text including refinements required to the simulation. Data was collected in real time, with observers following the simulated process and workflow being tested; video review was not used.

#### Study

The ‘Study’ component of the method allowed data to be captured from multiple sources and formats, both qualitative and quantitative in nature. This enabled precise recognition of system failure, at what point it occurred in the process, and often captured possible solutions to the identified error. Qualitative data included direct observation of the process testing, staff experience within the simulated process/workflow, and participant and simulated patient feedback during the debrief. Comparison studies of actual events to the written processes/workflows and to their anticipated outcomes occurred throughout the activity. Examples of quantitative data collected included emergency team response times, amount of time between unexpected patient presentation at the reception desk to ambulance arrival, and the length of time for ambulance transport from the regional hospital to the central health service.

#### Act

**Fig. 2:**
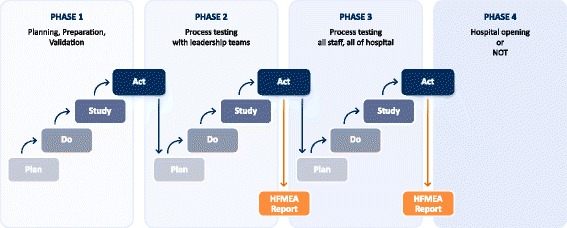
Phases of the simulation testing and the timing of the PDS(A) and HFMEA reports

The *‘*Act’ component of the PDSA framework is absent from the form because the identified actions were represented through the ‘HFMEA Summary Report’ document. The Plan, Do, Study, (Act) components of the PDSA framework informed the execution and iterative nature of the study. Figure 2 shows all the four phases of implementation and alignment of reporting structure.

### Simulation activity Phases 2 and 3: simulation-based quality improvement tool

Objectives:

1. Conduct the simulations
2. Utilise and revise the SQIOT
3. Collate and rate risks derived from collected data using the HFMEA Summary Report
4. Report results to organisational decision-makers

Phase 2 testing employed two large-scale iterations, with each iteration being 2 days in duration. Over the two iterations (4 days), a total of 13 simulations were undertaken, as outlined in Table 1. Simulations included unexpected emergency presentations to the front desk, ward-based emergencies, patient discharge processes, administration and housekeeping processes, and general patient care scenarios. The organisation’s leadership team were the participants and observers throughout Phase 2 and comprised of senior executives and clinical leaders (*n* = 14). During this phase, changes were made to the systems and workflows as a result of the simulations. Processes and the simulation scenarios were amended and refined. Any concerns or inefficiencies identified with the SQIOT were also reviewed. The overarching project utilised a participatory action-research model [23] that allowed the SQIOT form to be reviewed and refined between iterations of testing. Key improvements to the tool are outlined in Table 2.

**Table 2:** Changes made to SQIOT during testing

Amendments made to SQIOT	Phase of testing	Rationale
Document design	Phase 1	The document went through multiple versions upon commencement of simulations within the simulation centre and as the hospital processes and workflows were being redeveloped and amended.
Section: do number of steps in a process reduced	Amended after Phase 2 Test 1	Initially, the SQIOT form was too prescriptive as it outlined every single expected step in a care episode or process. As a result, the ‘Do’ section of the SQIOT was predominately not completed by observers during Phase 2.
Section: do addition of ‘Not Applicable’	Amended after Phase 2 Test 2	Data collectors expressed confusion when completing the tool because a number of prescribed equipment or processes were not in place. What was needed was an option of ‘N/A’ (not applicable) in addition to ‘Yes or No’.
Section: study increased space for free text	Amended after Phase 2 Test 1	Observers were a mixture of clinical experts, simulation team members, hospital leaders, service support staff, and the embedded simulated patients. After test one, observers requested additional free text space. The key benefit of adding more open space for comments was that observers could document their view, thus ensuring the process was evaluated from multiple perspectives. Later, during data analysis, it was possible to identify themes from the varied observer groups.

Phase 3, an intense 24-h event, occurred 3 weeks after Phase 2 and involved hospital staff from every service and department rostered over three shifts. Phase 3 consisted of 24 simulations, with trained community members participating as simulated patients (*n* = 12), hospital staff (*n* = 70), third party providers (*n* = 14) (e.g. ambulance, fire services, funeral directors), and MEST members (*n* = 7). All systems and departments operated in synchrony under live simulated conditions with both mannequin-based and simulated patient scenarios. Hospital staff were surveyed after each simulation activity via a validated tool to ascertain their confidence level in the system/process being tested [24]. At the completion of the 24 h, the hospital leadership, via a staff survey, sought the opinion of the staff on hospital readiness. In Phases 2 and 3 of testing, 67 SQIOT forms were completed.

### Simulation activity Phases 2 and 3: HFMEA Summary Report (Act)

**Fig. 3:**
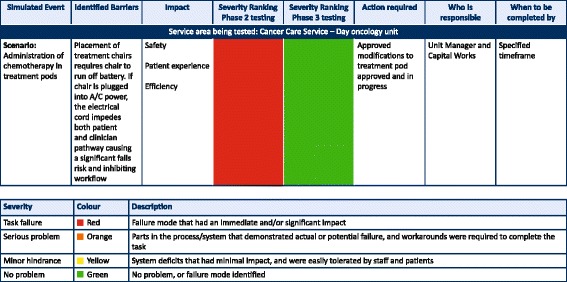
Sample from HFMEA Summary Report

The MEST responsibility was to test systems, analyse data, and to report the findings. MEST was not mandated to take accountability for identified concerns or to be accountable for rectifying system deficits. It was the responsibility of organisational leaders to call to action appropriate people or services, to address the identified concerns. This required designing an effective reporting tool, the ‘HFMEA Summary Report’. An extract can be seen in Fig. 3.

All documented data from each test and phase captured on the SQIOT was collated and presented in a format that highlighted the risks, identified potential system impacts, and allowed for decision-makers to prioritise future actions. HFMEA is normally used to identify causes and effects of failure modes in systems and processes before a significant event or near miss occurs [13, 14, 25, 26]. In this study, the HFMEA was used for simulated patient care activities in the real hospital environment. Health Failure Modes, Effects Analysis, and traditional Failure Mode Effects Analysis provide numerical ranking for the identified failures or risks based on severity and probability [13, 23, 25, 26]. Numerical ranking scales vary, e.g.1–16 [13, 14, 26], 1–10 [25], 1–5 [27], or 1–4 [28] typically with 1 being ‘no’ or ‘minor impact’ through to the largest number meaning catastrophic harm (e.g. permanent patient harm) [25, 26]. The methodology permits a specific process to be broken down into its elements and analysed for actual and potential weaknesses or failures within the process or system (failure modes) [13, 26]. Because this study generated a large amount of information, simplification was needed. The solution was (1) the risk ranking scale was comprised of a colour coding system (refer to Fig. 3 for the severity ranking matrix; (2) identified concerns/barriers were organised in accordance with the organisation’s five strategic priorities [22]; and (3) each identified failure mode was analysed to identify the likely cause. Once a failure mode was identified and reported, the clinical and non-clinical directors then assigned individual accountability to rectify identified concerns within specified time frames. This report format eased the burden on decision-makers to identify, prioritise action, and apply remedies to failure modes prior to errors reoccurring [12, 25].

The Phase 3 report combined both Phases 2 and 3 test results demonstrating where improvements had been made, or not made, with descriptions from each test iteration, i.e. something akin to a longitudinal study. The goal of the longitudinal descriptions was to assist the Commissioning Steering Committee to prioritise what it should and could rectify prior to live service delivery. In Table 3 are a few examples of failure modes identified during the simulated testing, alignment with organisational strategic priorities, and summary of associated outcomes.

**Table 3:** *Examples of identified failure modes* illustrate actual or potential failure modes

Failure mode (identified barrier)	Impact	Outcome
Position of A/C power cord to cancer care treatment chairs demonstrated significant falls risk to the patient and staff. Also inhibitive of efficient staff workflow	Safety Patient experience Efficiency	Each treatment pod had additional power point installed and TV’s repositioned in each pod
No standing orders for adrenaline administration in an emergency (no resuscitation team onsite)	Safety	Organisational Resuscitation Committee endorsed standing orders for the hospital for all registered nurses to administer adrenaline in a medical emergency
Imminent birth presenting to reception (no emergency department) No wheelchair or trolley at front reception to move patient to another location No neonatal resuscitation equipment available Unknown process—unknown if oxytocic medications are/will be available from pharmacy	Safety Efficiency Patient experience	Required equipment purchased and staff orientated to its location and function Pharmacy consulted and process implemented
Adult (chest pain) and a paediatric (asthma) presenting to front reception With one administration staff on front desk, cannot initiate required phone calls at the desk to get help and attend to patient Insufficient emergency equipment located on reception level of the hospital Support services assistant and security currently not on medical emergency page (security: external company) Staff knowledge in management of a paediatric emergency or first aid measures (no paediatric inpatients for this hospital)	Safety Efficiency Patient experience	Recommendations implemented: Staff assist button for front reception Mobile phone/deck phone at reception External security company to be added to the emergency pager list Emergency equipment purchased for reception: - Oxygen and masks (adult and paediatric) - AED - Vital signs monitor - PPE - IV cannulation Paediatric education plan and resuscitation training planned and commenced
Volume of overhead emergency buzzer in inpatient areas. Staff in single rooms with door closed cannot hear staff assist or code blue call bells	Safety Efficiency	Company contacted and volume increased

## Discussion

In the literature, there is a paucity of evidence of using PDSA QI as a methodology in healthcare simulation testing alongside HFMEA reporting. The methodology used in this study produced results that satisfied leadership and led to an uneventful opening of the satellite hospital with no patient or staff harm, and a grateful community.

The iterative amendments to the tool throughout Phases 1–3 allowed for continued improvements in data collection. Small changes such as reducing the number of steps documented in a process led to more data being collected and more meaningful data. This improvement was thought to be attributed to (1) reducing cognitive load for the data collectors, (2) better characterising of the ‘real-life’ processes, and (3) highlighting the salient parts of the process.

Observers were trained how to use the tool, but did not have an opportunity to practice using it in action prior to the commencement of Phase 2, Test 1. It seems likely that data collection would have been strengthened by observer training. Nevertheless, useful data was collected with each iteration and, as might be expected, the data usefulness improved as observers became more experienced, and the instruments designed with flexibility in mind were improved as the multi-phased testing process was implemented.

Although this activity was not testing a single system or process, it required the design of a report that could capture the actual and potential failure modes of multiple individual processes and systems. To do this effectively, the second template (HFMEA Summary Report) was designed. This documentation methodology proved beneficial in two ways. First, it provided organisational decision-maker’s precise and actionable information on identified latent failures with sufficient information to make informed decisions based on priorities and resourcing. Second, it served as positive reinforcement for middle management and front line staff that their concerns were being taken seriously by the senior managers and that their suggestions for improvement were acted upon. It reinforced a positive safety culture for which all staff felt empowered and invested in the quality and safety of their new hospital.

Normally, HFMEA identifies the points of failure from a process perspective. This study went a step further through the inclusion of feedback from the simulated patients as their role as consumers. A limitation of testing a system or process under simulated conditions is the validity of ‘guessing’ or making assumptions about the patient perspective as a recipient of care within that system. To help ameliorate this problem and strengthen the testing of the system and its interdependencies, local consumers who would be using the health service were trained as simulated patients. The simulated patient participated in the associated simulation activity debrief, and their thoughts and opinions were captured. This provided reassurance that a system or process was safe and efficient, while at the same time resulted in a positive patient experience. The idea was to combine safe and reliable processes with a patient-centred approach. Simulation and HFMEA used collaboratively permitted unique and precise prioritising failure modes. Without this comprehensive approach, failure modes might have remained unnoticed or unrecognised [14, 25, 29, 30].

The hospital leadership and Commissioning Steering Committee team realised the value of the simulation activity. This realisation led them to make accurate and well-informed decisions regarding operational readiness. They were so confident in the simulation-based testing process that a delay in the highly publicised opening date was among the possible options. Patient safety and staff preparedness was paramount. The simulation testing and the detailed reporting of the activities added strength to the decision-making of the hospital leadership team and provided reassurance to the hospital executive council, Commissioning Steering Committee, and the hospital board members.

## Conclusion

The simulation methodology utilised for the testing of health systems and processes provides a unique lens through which staff at all levels can observe, assess, and evaluate [1, 5–7]. The advantage of simulation as a quality improvement methodology is that one cannot accurately predict system performance without testing prior to ‘going live’. While we may intuitively agree with the truth of that statement, we and our health service were extremely pleased to see how much true value was received via the simulation-based testing and the associated methodology provided in this report. Smart, dedicated professionals created the physical layout, thoughtfully designed the systems, and placed highly trained professionals within these designs. The natural motivation of all these professionals was patient and staff safety, system effectiveness, and patient satisfaction. Despite all these talents and dedication, there were still a number of weaknesses and errors, a few of which with a pretty high likelihood of resulting in a costly error remained in the system. The PDSA methodology utilised within this iterative and increasingly complex simulation activity allowed incremental and substantial positive changes to occur. While the process does use a number of resources, it is believed to have resulted in a greater likelihood in producing more positive and widespread sustainable change in the organisation, than ‘one off’ change implementation [10, 31]. This study demonstrated that in this circumstance, an iterative PDSA quality improvement methodology was an effective framework for structuring simulation-based healthcare system testing. The PDSA and HFMEA frameworks collectively allowed rapid data collection regarding individual systems or processes, and a larger ecosystem working in synchrony during a 24-h simulation event. This allowed visibility of the interrelated parts and gaps within the coordination between individuals, departments, and systems.

The validity of the conclusions is fundamentally dependent on the accuracy of the simulation scenarios. The future use of this type of simulation testing needs to include how to portray clinical process authentically enough to elicit appropriate care delivery and staff behaviours and attitudes that would naturally occur during service delivery. In this activity, we believe that the quality of the simulations were sound to start with and got better with each iteration. Scenarios were designed and tested during Phase 1 within the simulation centre. Additionally, the simulations were run four times within the real hospital environment (Phases 2–3) with continuous revalidation of scenario content. Although there were multiple observers of the simulation activities, there may have been interactions or potential system weaknesses missed simply due to observers’ natural inability to see and hear all. Consideration for future activities should be capturing video of the simulations to undertake a second review and to enable inter-rater reliability studies of the Observation Simulation Quality Improvement Observation Tool.

The inclusion of feedback and suggestions from frontline staff ultimately led to better processes and less workarounds, as the staff helped to shape and design the system in which they would function. A secondary outcome was that before the doors of the health service opened, there was a positive simulation culture throughout the organisation, and recognition of how simulation methodology can support a health service beyond education and training.
